# Emerging Evidence for Platelets as Immune and Inflammatory Effector Cells

**DOI:** 10.3389/fimmu.2014.00653

**Published:** 2014-12-18

**Authors:** Matthew T. Rondina, Olivier Garraud

**Affiliations:** ^1^Division of General Internal Medicine, University of Utah, Salt Lake City, UT, USA; ^2^Program in Molecular Medicine, University of Utah School of Medicine, Salt Lake City, UT, USA; ^3^Department of Internal Medicine, George E. Wahlen Department of Veterans Affairs Medical Center, Salt Lake City, UT, USA; ^4^Faculty of Medicine of Saint-Etienne, University of Lyon, Lyon, France; ^5^French Blood Establishment, Auvergne-Loire, Saint-Etienne, France

**Keywords:** platelets, immunity, infection, sepsis, pathogens

## Abstract

While traditionally recognized for their roles in hemostatic pathways, emerging evidence demonstrates that platelets have previously unrecognized, dynamic roles that span the immune continuum. These newly recognized platelet functions, including the secretion of immune mediators, interactions with endothelial cells, monocytes, and neutrophils, toll-like receptor (TLR) mediated responses, and induction of neutrophil extracellular trap formation, bridge thrombotic and inflammatory pathways and contribute to host defense mechanisms against invading pathogens. In this focused review, we highlight several of these emerging aspects of platelet biology and their implications in clinical infectious syndromes.

## Introduction

Platelets are small anucleate cells highly specialized for hemostasis and vascular wall repair. Emerging data demonstrate that in addition to their traditional hemostatic functions, platelets are also versatile effector cells with a repertoire of functions that span the immune continuum. These newly recognized and well-established platelet functions bridge thrombotic and inflammatory pathways and contribute to many systemic inflammatory and immune processes and diseases (1–3). Moreover, the inflammatory and immune specializations of platelets are likely evolutionarily driven adaptations that augment host defenses against invading pathogens. These newly recognized platelet activities include the release of pleiotropic immune mediators, heterotypic interactions with endothelial cells, monocytes, and neutrophils, toll-like receptor (TLR) mediated responses, and induction of neutrophil extracellular trap (NET) formation. Here, we provide a focused review on several of the emerging aspects of the biology of platelets across the inflammatory and immune continuum. For additional information on these topics, the reader is referred to several recent reviews (1, 3–5).

## Platelet Surface Ligands Sense and Respond to Pathogens

**Figure 1:**
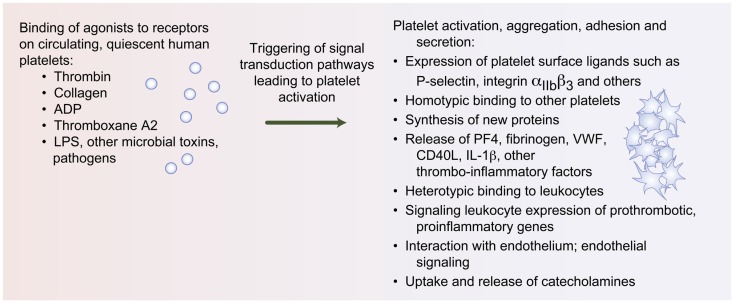
**Upon activation, platelets mediate responses central to inflammation and hemostasis**. Agonists such as thrombin, collagen, adenosine diphosphate (ADP), thromboxane A2, and LPS bind to agonists on human platelets, triggering classic responses of platelet activation, aggregation, adhesion, and secretion. Platelet activation also results in protein synthetic activities, release of thrombo-inflammatory modulators, heterotypic binding to leukocytes, and interaction with the endothelium.

The platelet surface is replete with numerous receptors that not only regulate hemostatic responses but also trigger proinflammatory and immune cascades (Figure 1). Many of these surface receptors have been called “immunoreceptors” in homage of their molecular structure and the ligands they recognize (6). For example, platelet Fc receptors bind immunoglobulins of the IgE, IgG, and IgA class and immune complexes, directly inducing immune signaling pathways. Glycoprotein VI (GPVI), which is only found on platelets, triggers platelet microvesicle release and subsequent inflammatory signals through interleukin (IL)-1 (7). GPVI may also be a receptor for hepatitis C, mediating viral transport and replication mechanisms (8). Moreover, GPVI amplifies platelet activation by thrombin, thus providing a mechanism for coordinate signaling with G-protein-coupled pathways (9). C-type lectin-like (CLEC-2) receptors, including DC-SIGN, mediate human immunodeficiency virus 1 (HIV-1) capture by platelets (10) and platelet activation in dengue infection (11).

Human platelets and megakaryocytes also express mRNA and/or protein for the TLRs 1, 2, 4–7, and 9 (2, 12–15). TLRs bind diverse ligands from many infectious pathogens, including bacteria, viruses, parasites, and protozoa. Thus, the discovery that human platelets possess TLRs established direct mechanisms by which platelets may function as pathogen “sensors” (12, 16). Moreover, by recognizing endogenous ligands as well as microbial pathogen-associated molecular pattern motifs, platelet TLRs provide pathways by which human platelets can response to danger associated molecular patterns (DAMPs), whereby mediating both infectious and non-infectious immune syndromes (17, 18).

**Figure 2:**
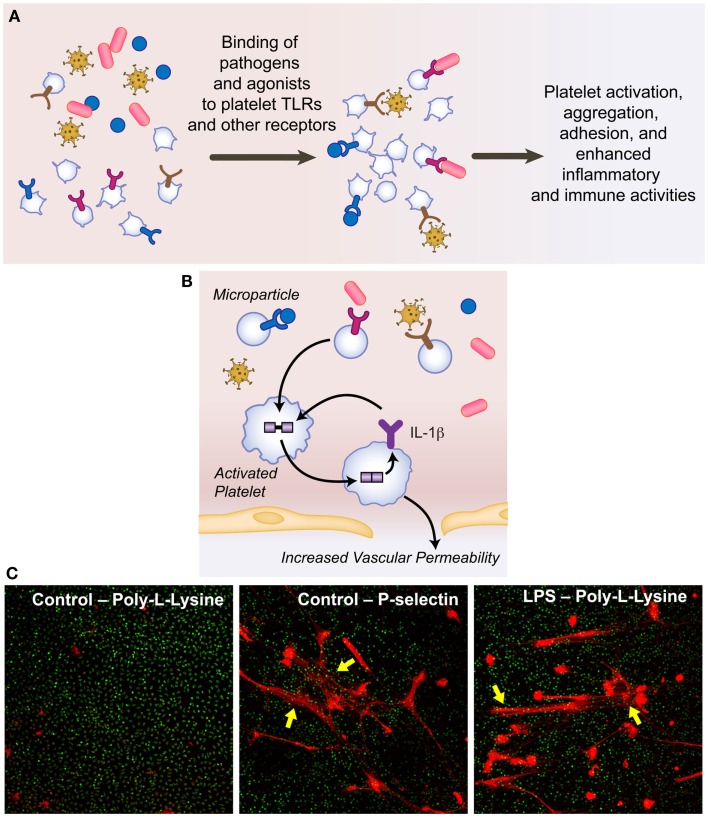
**Platelets interact with pathogens present in the infections milieu, increase vascular permeability, and mediate NET formation**. **(A)** Binding of pathogens and agonists present in the infectious milieu leads to platelet activation and enhanced inflammatory and immune activities. **(B)** Platelet-derived microparticles carry IL-1β, which increases vascular permeability. **(C)** P-selectin, which is expressed on the surface of activated platelets, induces NET formation in a reductionist model of endothelial cell and platelet activation. PMNs isolated from healthy adult donors were incubated on poly-l-lysine or p-selectin coated glass coverslips and assessed qualitatively for NET formation using live cell imaging. Extracellular, NET-associated DNA is shown in red fluorescence (Sytox Orange) and intracellular, nuclear DNA is shown in green fluorescence (Syto Green). NET formation by LPS-stimulated PMNs is shown as a positive control. These images are representative of four separate experiments performed using PMNs isolated from four different healthy adult donors (used with kind permission from Drs. Nathan L. Thornton and Christian C. Yost).

Among the TLRs identified on platelets at either the mRNA and/or protein level, TLR4 has been most extensively studied to date (2, 12). TLR4 is the receptor for lipopolysaccharide (LPS), an endotoxin component on the outer membrane of Gram-negative organisms that elicits strong immune responses. LPS activates human platelets both *in vitro* and during *in vivo* settings where healthy volunteers are given low doses of LPS, although activation patterns are variable (19–22). Although still incompletely understood, LPS-induced platelet responses include aggregation, degranulation, secretion, pre-mRNA splicing and protein synthesis, and alterations in surface CD40L. Moreover, LPS-induced platelet activation triggers formation of NETs by polymorphonuclear leukocytes (PMNs), enhancing bacterial capture, and killing (23) (Figure 2).

Emerging data have identified new and intriguing roles for platelet TLR7 during viral infections. TLR7, which localizes to the endosomal compartment of the cell (24), is a sensor for nucleic acid ligands – in particular for ssRNA. Viral pathogens such as influenza and HIV, which continue to have significant public health burden, activate the TLR7 receptor (24). These and other ssRNA viruses, which signal through TLR7, are associated with thrombocytopenia, giving rise to suppositions that platelet TLR7 mediates viral sensing and/or host defense mechanisms. In recent and elegantly performed studies (15), activation of platelet TLR7 not only induced platelet-neutrophil aggregation and a reduction in platelet count, but was also necessary for optimal survival in murine models of encephalomyocarditis (EMCV) viral infection. Engagement of platelet TLR7 under these conditions, however, did not induce platelet aggregation (15). Thus, platelet TLR7 plays a key role in host recognition of ssRNA viruses and suggests that platelets may be integral mediators of innate immune responses.

Toll-like receptor9, while less well examined currently, is intriguing given its unexpected cellular localization patterns (19, 25). TLR9 is expressed on the plasma membrane and in the cytoplasm of quiescent human platelets and its surface expression increases upon activation of platelets with agonists such as thrombin (14, 25, 26). TLR9 is a ligand for unmethylated CpG islands in viral and bacterial DNA, suggesting a previously unrecognized system of pathogen sensing by human platelets (Figure 2). Moreover, TLR9 binds a carboxyalkylpyrrole protein adduct that may serve as a DAMP, resulting in platelet activation, aggregation, and granule secretion and thrombosis (27). Thus, TLR9 represents another functional platelet receptor linking immune, inflammatory, and thrombotic pathways.

## Platelets Secrete Mediators that Augment Host Immune Mechanisms

Activated platelets have numerous direct and indirect mechanisms for delivering signals to target cells involved in immune and inflammatory interactions (Figure 1). These diverse mechanisms include platelet secretion of chemokines, cytokines, and other mediators (4). There are more than 300 proteins secreted by platelets, the majority of which are stored in at least one of three types of storage organelles: alpha granules, dense granules or bodies, or lysosomes. Recently, a fourth electron-dense tubular system compartment, called the T-granule, was discovered and, intriguingly, co-localizes with TLR9 (25). Many of these proteins translocate to the platelet surface and are released upon platelet activation. Other secreted proteins are located basally on the platelet membrane and/or within other subcellular stores (Figure 1).

Protein products released by platelets orchestrate intercellular signaling for dynamic immune and inflammatory response (1, 2, 4). For example, quiescent human platelets possess the IL-1β pre-mRNA but almost no mature IL-1β mRNA or IL-1β protein (28). Upon activation with agonists often present in the infectious milieu, including thrombin and LPS, platelets synthesize pro-IL-1β protein (28, 29). Components of the inflammasome, which regulates host defense mechanisms to pathogens (30, 31), are present within platelets and mediate conversion of pro-IL-1β to the mature IL-1β cytokine (2). Mature IL-1β, the gatekeeper of inflammation, can be released by platelets into the systemic circulation and/or packaged within platelet-derived microparticles (MPs). IL-1β within platelet MPs allows for communication with and activation of extravascular cells and induction of endothelial permeability (7, 32). Recent evidence demonstrates that IL-1β also acts through an autocrine loop to amplify platelet activation responses (33). Platelet shedding of IL-1β rich MPs also may contribute to enhanced vascular permeability in hemorrhagic viral infections such as dengue (34) (Figure 2). Other proteins secreted by platelets, including platelet factor 4 (PF4), RANTES (*r*egulated on *a*ctivation, *n*ormal *T*-cell *e*xpressed and *s*ecreted), CD154, transforming growth factor beta 1 (TGF-beta 1), and CD40L are involved in key pathways for innate immune regulation, inflammation, and adaptive immune responses (1, 19, 35–38).

## Clinical Implications of Platelet Immune Activities

Emerging and established evidence demonstrates that platelets participate in diverse inflammatory and immune clinical syndromes. While a detailed discussion is beyond the scope of this review, the reader is referred to several recent articles summarizing these activities (1–3, 12, 19, 36, 37). In the section below, we briefly discuss several of these clinical conditions.

Platelets are key mediators of bacterial and viral infectious syndromes. Emerging and intriguing data also highlight that fungal pathogens such as invasive aspergillosis, also induce platelet activation *in vitro* and *in vivo*. Moreover, upon activation by fungal serine protease and the mycotoxin gliotoxin, platelets inhibit fungal growth (39). These and other data demonstrate that platelet-fungus interactions are multifaceted and complex, involving complement activation, chemotactic attraction, and activation of other immune cells such as phagocytes (40).

Among these various infectious syndromes, sepsis, malaria, and dengue are particularly important to highlight given their commonness and high risk of significant morbidity and mortality (41). Alterations in platelet number and function are integral to each of these, yet the mechanisms underlying these changes remain only incompletely defined. Moreover, the extent to which platelets augment host defense mechanisms or, contrarily, induce injurious systemic responses is unclear.

Thrombocytopenia is common in septic syndromes with clinical studies demonstrating an inverse correlation between platelet number and adverse outcomes (12, 42, 43). The precise mechanisms underlying reductions in platelet counts during infectious syndromes are not entirely clear although interferon production during infections may contribute to impairments in thrombopoiesis and resulting thrombocytopenia. The precise pathways by which interferon causes an antiproliferative effect are still incompletely understood but likely involve direct effects on megakaryocytes residing within the bone marrow niche.

While many studies have focused on alterations in “traditional” platelet activation patterns (44, 45) (e.g., adhesion, secretion, platelet-monocyte aggregation), emerging evidence highlights the new biology of platelets in septic syndromes. For example, in response to *in vitro* stimulation LPS, thrombin, bacteria, and bacterial toxins, human platelets process constitutively present tissue facture (TF) pre-mRNA, resulting in a mature TF transcript, synthesis of TF protein, and generation of TF-dependent procoagulant activity (22). Moreover, platelets from septic patients, but not healthy controls, express the mature, spliced TF mRNA, demonstrating that the septic milieu may alter the platelet transcriptome. Preliminary analyses of next-generation RNA sequencing data (“deep sequencing”) performed on platelets from septic patients identified numerous transcripts that are significantly altered compared to matched, healthy control subjects. Many of these altered transcripts mediate key immune and inflammatory pathways and augment host defenses to bacterial invaders.

Dengue, a mosquito-borne viral infection, has variable clinical manifestations. Nevertheless, especially in more severe cases, marked thrombocytopenia, hemorrhage, “capillary leak,” and shock may occur. While the dengue flavivirus targets endothelial cells and leukocytes, interactions of the virus with platelets and megakaryocytes is likely a central feature of the pathophysiology and course of infection (41). Cytokines synthesized by platelets or in response to platelet-dependent signaling of monocytes, including IL-1β, IL-8, tumor necrosis factor (TNF)-α, and monocyte chemotactic protein (MCP)-1 (19), are increased in plasma from dengue patients and correlate with the degree of thrombocytopenia (46). Increased IL-1β in platelets and platelet MPs was recently reported to occur through a mechanism dependent on mitochondrial reactive oxygen species (ROS)-triggered NLRP3 inflammasomes (34). Dengue virus also alters l-arginine transport and nitric oxide generation in platelets *in vitro*, resulting in impaired aggregation (47). Platelets isolated from dengue patients have evidence of mitochondrial dysfunction with activation of apoptosis pathways, mediated through DC-SIGN (11). These findings suggest that platelet dysfunction may contribute to the thrombocytopenia and associated hemorrhagic complications of dengue infection.

Platelets are also likely early responder and effector cells in malaria infections. Human platelets inhibit the growth of some malaria species and also kill the malarial pathogen in parasitized red blood cells (PRBCs), experimental evidence that platelets may serve as early host defense responses to malarial infection in the vasculature (48–50). In contrast to these protective innate immune activities, platelets may also contribute to injurious vasculopathy in severe malaria. While the mechanisms are still not entirely understood, murine studies demonstrate that platelet signaling via PF4 release mediates T-cell recruitment and monocyte activation within the central nervous system (CNS) (51). These processes may contribute to CSN injury in severe malaria. Recent studies of patients infected with *Plasmodium vivax* malaria suggest that the thrombocytopenia that commonly occurs in malaria may be due to platelet phagocytosis (52). Taken together, these studies and others provide experimental and clinical evidence that platelets are important immune effector cells in malaria (53, 54).

## Conclusions

While traditionally thought of as primary hemostatic cells, platelets are emerging as dynamic effector cells that sense and respond to invading pathogens. Ongoing studies in experimental models of infection and human studies will further elucidate the mechanisms, pathways, and processes governing the platelet’s broad repertoire of immune functions. These previously unrecognized findings may also lead to the development of novel therapies targeting the platelet to augment host defense mechanisms.

## Authors’ Contributions

Each author contributed to the manuscript ideas and content. Each author was involved in writing the manuscript, the decision to submit the manuscript for publication, and final editing and approval.

## Conflict of Interest Statement

The authors declare that the research was conducted in the absence of any commercial or financial relationships that could be construed as a potential conflict of interest.

## Abbreviations

CLEC-2, C-type lectin-like 2; CNS, central nervous system; DAMPs, danger associated molecular patterns; GPVI, glycoprotein VI; HIV-1, human immunodeficiency virus 1; IL, Interleukin; LPS, lipopolysaccharide; MCP-1, monocyte chemotactic protein-1; MPs, microparticles; NET, neutrophil extracellular trap; PF4, platelet factor 4; PMNs, polymorphonuclear leukocytes; RANTES, regulated on activation, normal T-cell expressed and secreted; ROS, reactive oxygen species; TLR, toll-like receptor; TNF-α, tumor necrosis factor alpha.
